# Appropriate Donor-Acceptor Phase Separation Structure for the Enhancement of Charge Generation and Transport in Polymer Solar Cells

**DOI:** 10.3390/polym10030332

**Published:** 2018-03-18

**Authors:** Dayong Zhang, Rong Hu, Jiang Cheng, Yuqiang Chang, Mingming Huo, Junsheng Yu, Lu Li, Jian-Ping Zhang

**Affiliations:** 1 State Key Laboratory of Electronic Thin Films and Integrated Devices, School of Optoelectronic Science and Engineering, University of Electronic Science and Technology of China (UESTC), Chengdu 610054, China; zdy_93@126.com (D.Z.); jsyu@uestc.edu.cn (J.Y.); 2 Research Institute for New Materials Technology, Chongqing University of Arts and Sciences, Chongqing 402160, China; jiangcheng@cqwu.edu.cn; 3 Department of Chemistry, Renmin University of China, Beijing 100872, China; xiaolin-xiaosun@163.com (Y.C.); jpzhang@chem.ruc.edu.cn (J.-P.Z.); 4 Qingdao Research Center for Advanced Photonic Technologies, Laser Research Institute, Shandong Academy of Sciences, Qingdao 266100, China; huo_mingming@126.com

**Keywords:** polymer solar cells, PC_71_BM acceptor, PTB7 donor, DIO additive, charge generation and transport, phase separation structure

## Abstract

The morphology of active layer for polymer solar cells is critical to enhance the performance especially for fill factor of the devices. To investigate the relationship between active layer morphology and performance of polymer solar cells (PSCs), 1,8-diiodooctane (DIO) additive, and [6,6]-phenyl-C_71_-butyric acid methyl ester (PC_71_BM) electron acceptor were used to regulate the aggregation morphology of copolymer poly(thieno[3,4-b]-thiophene/benzodithiophene) (PTB7) electron donor from solution state to solid state. Atom force microscopy (AFM), steady-state absorption (UV-Vis), time-resolved absorption (TA), spectroelectrochemistry (SEC) and current-voltage (J-V) measurements were employed to characterize the morphology, optical and electrical characteristics of active layers and to reveal the relationship among the morphology, photophysical property, and performance of PTB7-based devices. The results show that DIO can refine the aggregation scale of PTB7 during the dissolution process, whereas both the aggregation scale and aggregation behaviors of PTB7 donor are affected by PC_71_BM acceptor molecules. Furthermore, the bulk heterojunction structure (BHJ) morphology of active layer can be optimized during the DIO evaporation process. TA kinetic data indicate that the population and lifetime of charged species are improved in the DIO-treated BHJ active layer. Moreover, the active layers with DIO treatment have a relative low highest occupied molecular orbital (HOMO) energy level, which makes hole transport more easily in PTB7 donor phase. As a result, the performance of PTB7-based PSCs is enhanced.

## 1. Introduction

Polymer solar cells (PSCs) are one of hot option for solving the problem of energy shortage owing to their advantages in flexibility, large-area, solution-processed [1,2,3]. Recently, over 10% power conversion efficiency (PCE) has been reported for bulk heterojunction (BHJ) devices based on binary, ternary or tandem structures [3,4,5], which making PSCs have great potential prospect of application. However, some disadvantages of PSCs such as poor stability, short service life and low PCE need to be realized compared with silicon-based solar cells. Hence, more effort needs to be paid for PSCs to reach the standard of commercial application.

Most of studies prove that the optimized morphology of active layer has an important influence on the performance of PSCs. Normally, a series of methods about solution preparation [6], film fabrication [7] and post-processing [8] are usually employed to regulate the BHJ morphology of active layer, since photogenerated exciton dissociation and charge transport are highly efficient in the optimized morphology of active layer [9]. The fabrication process has an important effect on morphology of active layer. For instance, Srinivasan et al. evaluated the performance of poly(thieno[3,4-b]-thiophene/benzodithiophene) PTB7:PC_71_BM solar cells by spin and spray coating fabrication methods. The spray-coating devices with more uniform active layer improved the charge transport and exhibited higher performance than spin-coating devices [10]. Yu et al. optimized the morphology of active layer from various parameters of spin speed and spin time to obtain the optimal device performance [11]. In addition, many research results showed that ordered aggregation degree and donor-acceptor (D-A) phase separation scale of polymer: fullerene, could be optimized via thermal annealing or solvent vapor annealing treatment [12,13]. These post-processing methods also increased the mobility of charge carrier and reduced the probability of charge recombination. However, for poly[4,8-bis-substituted-benzo[1,2-b:4,5-b′]dithiophene-2,6-diyl-alt-4-substituted-thieno[3,4-b]thiophene-2,6-diyl] PBDTTT and PTB7 electron donor systems, the morphology of these active layers was deteriorated by solvent vapor annealing or thermal annealing, resulting in a poor device performance [8]. Therefore, the addition of high boiling point solvent additive into precursor solution becomes an effective way to regulate the morphology of active layer. Agostinelli et al. adopted 1, 8-octanedithiol as additive to control the crystallization of polymer and continuous D-A interpenetrating network structure in Poly[2,6-(4,4-bis-(2-ethylhexyl)-4H-cyclopenta[2,1-b;3,4-b′]-dithiophene)-alt-4,7-(2,1,3-benzothiadiazole)] PCPDTBT:PC_71_BM polymer system [14]. Park et al. employed DIO as the additive improved the degree of aggregation and nanometer-scale phase separation of PTB7:PC_71_BM active layer [15]. These researches showed that the additive treated active layer contributed to separation transport for electrons and holes, and avoided the charge recombination obviously. Currently, the popular explanation for the ability of additives regulation the morphology of active layer is as follows. Additive can selectively dissolve donor material or acceptor material in solution, and the evaporation of the additive is slower than that of main solvent, which is beneficial to the formation of vertical phase separation structure during film-forming process. The morphology changes of copolymer induced by additive and accepter would affect the photophysical process of the PSCs device [16]. For instance, Szarko et al. revealed the great variation of PTB7 excited state lifetime in solution, pure film and D-A BHJ blend film. The difference is mainly due to the strong π-π interaction and D-A interface structure [17]. Sharma et al. used transient spectroscopy to investigate the effect of ICBA on charge photogeneration dynamic process of PTB7:PC_71_BM, and it was found that the addition of ICBA seriously worsens the charge separation and transport [18]. Despite many literatures have studied the relevance between the morphology of active layer and the performance of PSCs device, as for solution-processed PSCs, the study on the relationship among solution state, solid state, photophysical property, and performance is rarely related based on PTB7-based system. Moreover, the effect of solvent additive treated precursor solution and active layer on the photoelectric conversion behavior needs more illumination.

**Figure 1:**
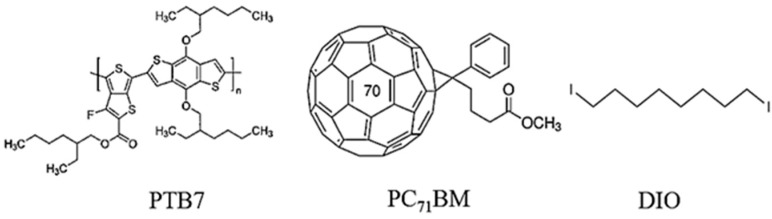
Chemical structures of PTB7, PC_71_BM and DIO.

In this work, to study the effect of DIO additive on the morphology and photoelectric property of polymer active layers, four kinds of PTB7-based devices were fabricated by spin coating method (cf. Experimental section), and Figure 1 shows the chemical structures of PTB7, PC_71_BM and DIO. To further understand the relationship between the morphology and performance of PSCs, optical characterization, including steady-state absorption, time-resolution absorption, spectroelectrochemistry, and morphology characterization methods were used to analyze the photoelectric behaviors in the varied PTB7-based active layers. More concretely, it was related to excited state dynamics process and charge generation and transport behaviors in donor polymer phases, which were regulated by DIO and PC_71_BM. Hence, the improved performance of PTB7-based PSCs could be illustrated. The result showed that both DIO and PC_71_BM had important effect on the aggregate state of PTB7 in solution and solid states, leading to a significant improvement in charge generation and transport. Thus, this work can afford a reference to optimize the structure and performance of PSCs, and provide a deep insight of photoelectric conversion process in PSCs.

## 2. Experimental

### 2.1. Fabrication of PSCs Devices

PTB7, PC_71_BM and DIO were purchased from Solarmer and Aladdin, respectively. The structure of PSCs was constructed by inverted configuration, i.e., indium tin oxide (ITO) substrate/zinc oxide (ZnO)/photoactive layer/molybdenum oxide (MoO_3_)/Ag electrode. The ITO substrate was successively washed with detergent, deionized water, acetone, ethanol and isopropyl alcohol, and then dried in a dry heat oven. To obtain ZnO electron transport layer, 60 μL precursor solution, which contained zinc acetate:2-methoxyethanol:ethanolamine (1 g:1 mL:0.28 mL) was spin-coated (3000 rpm, 30 s) on ITO substrate and followed by annealing 60 min on the heating plate at 200 °C. In this work, four types of active layers were also fabricated by spin-coating method. According to the researches before, most of solutions were consist of 97 vol% chlorobenzene (CB) and 3 vol% DIO, which is the best proportion for PTB7:PC_71_BM system [10,15,18]. The precursor solutions of active layers were PTB7 solution (9 mg mL^−1^, CB), PTB7 DIO + CB solution (9 mg mL^−1^, CB:DIO = 97%:3%, by volume), PTB7:PC_71_BM solution (9 mg mL^−1^:13.5 mg mL^−1^, CB), PTB7:PC_71_BM DIO + CB solution (9 mg mL^−1^:13.5 mg mL^−1^, CB:DIO = 97%:3%, by volume), respectively. The preparations of all active layers were conducted in a glove box filled with nitrogen. Accordingly, we named the four active layers as PTB7, DIO-treated PTB7, PTB7:PC_71_BM, and DIO-treated PTB7:PC_71_BM, correspondingly. After that, a thickness of 8 nm MoO_3_ hole transport layer and 50 nm Ag electrodes were successively deposited on the surface of the active layer using a shadow mask to obtain the effective area (0.07 cm^2^) of the devices and form a top anode.

### 2.2. J-V Measurement and EQE Measurement

The current density-voltage (J-V) curves of the devices were carried out on a computer controlled Keithley 2400 source meter. A xenon light source was used to provide an irradiance of 100 mW cm^−2^ (one sun at AM 1.5) at the surface of the devices. The external quantum efficiency (EQE) of device was measured in air at room temperature by using solar cell spectral response measurement system (SOFN Instruments Co. Ltd., Beijing, China).

### 2.3. Optical and Structure Characterization

The absorption of the active layers was studied by an UV-Vis-NIR spectrophotometer (Cary5000, Agilent, Santa Clara, CA, USA). The thickness of the active layers was 100 nm as determined with an Alpha-Skep surface profiler (KLA-Tencor, Milpitas, CA, USA). The morphology of neat and the blend active layers were characterized by atomic force microscopy (AFM, AFM-5500, Agilent).

### 2.4. Spectroelectrochemistry Test (SEC) and Time-Resolved Absorption Measurements (TA)

SEC tests of active layers were carried out with a classic three-electrode configuration consisting of a working electrode (active layer/ITO), a counter electrode (platinum wire) and a reference electrode (Ag^+^/Ag, AgNO_3_ (0.01 M) in acetonitrile), which were assembled in a special quartz cell with an effective optical path length of 2 mm. The electrolyte solution for electrochemical oxidation was tetra-n-butylammonium hexafluorophosphate (Bu_4_NPF_6_, 0.1 M) in acetonitrile. The electrical potentials across the reference and the working electrodes was 0.8 V. A potentiostat with ±2 V was employed as a power provider. The electrochemical cell was mounted on a UV-Vis-NIR spectrophotometer (Cary5000, Agilent). The spectroelectrochemical spectrum was taken as the difference between the absorption spectra at the equilibration time of oxidation and the background spectra without any biased potential. The detailed characterization of near-infrared time-resolved absorption (TA) for the active layer was described in literature [19].

## 3. Results and Discussion

### 3.1. Photovoltaic Performance of PTB7-Based Devices

**Figure 2:**
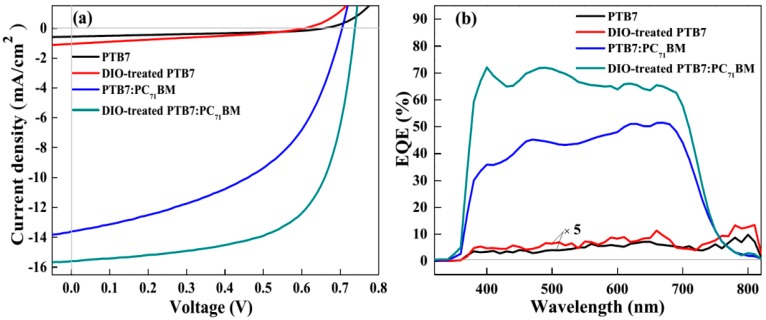
(**a**) J-V curves of PSCs based on varied active layers under the illumination of AM 1.5 G (100 mW cm^−2^); (**b**) EQE spectra of PSCs.

To investigate the effects of film-forming process and D-A structure on the performance of PTB7-based devices, four devices with varied active layers were fabricated (cf. Section 2.1). Their photovoltaic properties and parameters are shown in Figure 2a and Table 1, respectively. In the case of PTB7 device, it shows a low short circuit current density (J_sc_, 0.53 mA cm^−2^), open circuit voltage (V_oc_, 0.64 V) and fill factor (FF, 36%), resulting in a low PCE of 0.13%. As for DIO-treated PTB7 device, the J_sc_ and PCE are increased to 0.73 mA cm^−2^ and 0.17%, even a slight decrease of V_oc_ and FF. This indicates that DIO has advantages in improving the morphology of the PTB7 and enhancing the yield of photocurrent. In PTB7:PC_71_BM device, all photovoltaic parameters, including J_sc_ (13.44 mA cm^−2^), V_oc_ (0.70 V) and FF (48.5%) have been greatly enhanced compared with that of PTB7 device. Hence, a PCE of 4.52% is obtained. This phenomenon suggests that D-A structure could provide enough driving force for photogenerated exciton dissociation and charge transport [20]. Notely, the J_sc_ of DIO-treated PTB7:PC_71_BM device is 21 times and 1.2 times higher that of DIO-treated PTB7 device and PTB7:PC_71_BM device, respectively. Moreover, the V_oc_, FF and PCE of this device are also enhanced to 0.71 V, 64.7% and 7.30%. These results show that both DIO and D-A structure have important influence during film-forming process of active layer and performance of PTB7-based devices. To further analyze the effect of morphology on the enhancement of photocurrent, we measured EQE of neat PTB7 and BHJ PTB7:PC_71_BM devices. As seen from Figure 2b, the EQE spectrum of PTB7 device is rather weak, only a maximum value of 2% at 650 nm. Then, it has a slight increase to 2.5% in DIO-treated PTB7 active layer, which is in consistence with the tendency of J_sc_. Interestingly, the EQE is prominently enhanced by PC_71_BM phase at wavelength region of 340–800 nm, which has a maximum value of 51% at 673 nm. This indicates the yield of charge generation can be affected by PC_71_BM phase in BHJ devices. Moreover, the EQE is further improved after the PTB7:PC_71_BM active layer treated with DIO, suggesting that D-A phase structures are optimized for better charge generation and transport.

### 3.2. Steady-State Absorption Spectra of PTB7-Based Active Layers

**Figure 3:**
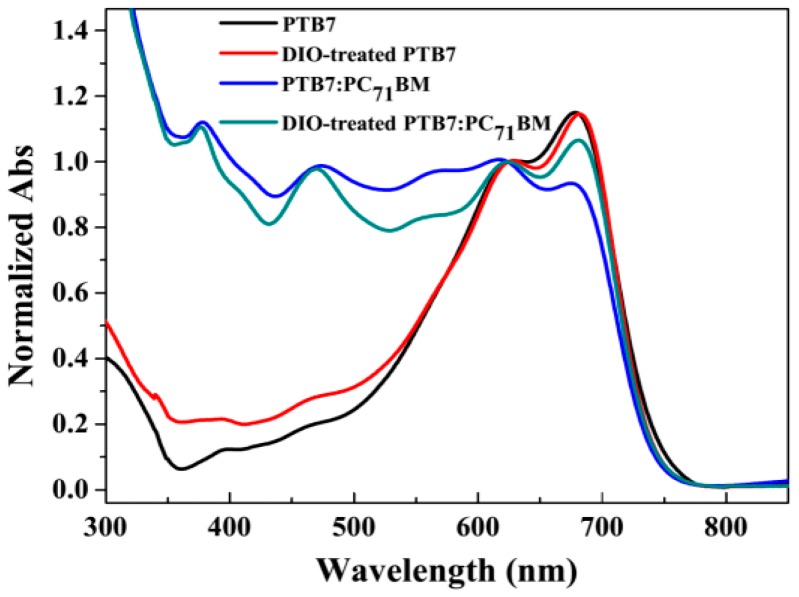
UV-Vis absorption spectra of PTB7-based active layers. All spectra were normalized at 623 nm.

In BHJ PSCs, the active layer is a core component of devices, which plays a role of converting photon energy to electrical energy [21]. Therefore, the steady-state absorption spectra of four active layers are measured by UV-Vis-NIR spectrophotometer, and the results are shown in Figure 3. As for the neat PTB7 active layer, main absorption band exhibits in the range of 400–750 nm, which are attributed to π-π* electron transition [22]. Moreover, two significant absorption peaks emerge at 623 nm and 678 nm, respectively. Based on the previous work [23], the longer wavelength absorption peak was attributed to the 0′-0 vibronic absorption, whereas the shorter wavelength absorption was due to the 1′-0 vibronic absorption. Herein, the sharpness and amplitude of 0′-0 vibronic absorption usually represent a metric of copolymer crystallinity or planarity. After adding 3% (volume ratio) DIO into PTB7 precursor solution, the relative absorption amplitude in the wavelength range of 400–550 nm is enhanced, presenting that the optical absorption capacity of the DIO-treated PTB7 device is improved. Additionally, compared with PTB7 active layer, the 0′-0 vibronic absorption peak has a slight redshift to 683 nm, which implies the crystallization of copolymer can be regulated by DIO solvent. In Figure 3, the absorption amplitude has a significant change in a wavelength range of 300–600 nm when PC_71_BM acceptor phase was mixed with PTB7 donor. Apparently, the characteristic peaks of PC_71_BM locate at 377 nm, 470 nm and 562 nm (Figure S1). Also, an important phenomenon can be ascertained that the amplitude ratio of 0′-0 and 1′-0 in PTB7:PC_71_BM active layer (0.93:1) is less than that in PTB7 neat active layer (1.15:1). This result indicates that PC_71_BM phase weakens the crystallization of PTB7. However, in DIO-treated PTB7:PC_71_BM active layer, the amplitude ratio of 0′-0 and 1′-0 increase to 1.07:1, which means that the DIO solvent still has a regulation effect on the crystallization of PTB7 even PC_71_BM-doped in the active layer.

### 3.3. Morphological Characterization of PTB7-Based Active Layers

**Figure 4:**
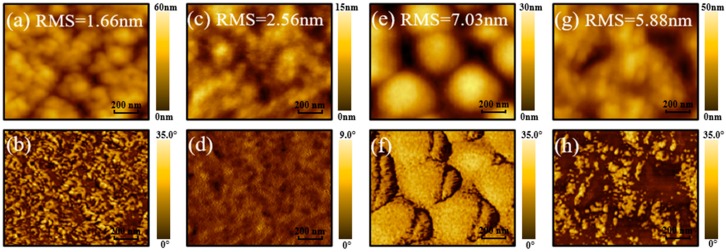
Representative AFM topography (**up**) and phase (**down**) images for PTB7-based active layers. (**a**,**b**) PTB7, (**c**,**d**) DIO-treated PTB7, (**e**,**f**) PTB7:PC_71_BM, (**g**,**h**) DIO-treated PTB7:PC_71_BM, the active layer was dried under the vacuum level of 10^−5^ Pa for 12 h.

To characterize morphology of PTB7-based active layers, AFM characterization was carried out by using tapping mode. Both topography and phase images of active layers are shown in Figure 4. As for PTB7 film, the surface morphology of active layer is rather smooth, which with a root-mean-square (RMS) roughness of 1.66 nm, and its phase image shows very weak phase structure (polymer-polymer phase). However, the active layer was spin-coated with PTB7 precursor solution with 3 vol % DIO, the RMS of DIO-treated PTB7 active layer is changed to 2.56 nm. The reason for this phenomenon is that DIO additive has high boiling point (167–169 °C), so the film forming process of PTB7 with DIO is slower that of PTB7 without DIO, and more PTB7 ordered aggregation form during this slow dry process. Meanwhile, phase image as shown in Figure 4c also authenticates that ordered aggregation degree of DIO-treated PTB7 active layer is improved compared to PTB7 active layer. In Figure 4e,f, the PTB7:PC_71_BM blend film without DIO additive exhibits coarse separation between PTB7 and PC_71_BM phase, which with 200–300 nanometer-sized domains and its RMS is determined as 7.03 nm. This D-A interface provides a driving force for exciton dissociation. In contrast, the DIO additive treated phase scale of BHJ active layer becomes more refined (RMS = 5.88) at the condition of slow dry process, and the phase scale is optimized to ~100 nm. By comparing with the J_sc_, V_oc_, FF of BHJ PTB7:PC_71_BM devices without and with DIO treatment, the improved PCE of DIO-treated device is mainly attributed to J_sc_ and FF. Hence, the additive regulation for BHJ active layer morphology is helpful for charge generation and charge recombination reduction.

### 3.4. Effect of Concentration, PC_71_BM and DIO Additive on Aggregation State of PTB7 in Solution

**Figure 5:**
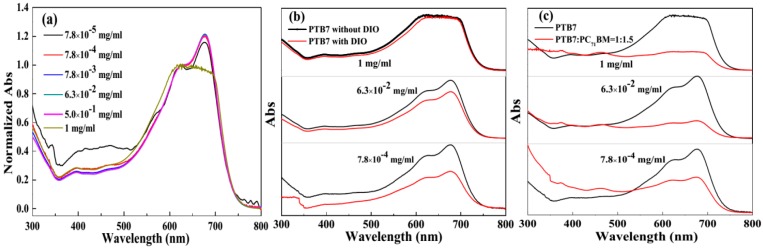
Steady-state absorption spectra of PTB7 solution and mixed PTB7:PC_71_BM solution at varied concentration conditions. (**a**–**c**) represent the effects of concentration, DIO additive and PC_71_BM on the steady-state absorption spectra of PTB7 solutions.

Solution-processed method is an advantage for the preparation of PSCs, both copolymer and fullerene derivatives or non-fullerene materials are mixed dissolved in organic solvent. According to previous literature [24], the conformation of the copolymer in solution-phase can be remained in solid-phase during the formation of active layer. That is to say, the state of the copolymer in solution-phase has an important effect on the morphology of active layer and the performance of devices. Based on this, we investigate the effect of concentration, DIO additive and PC_71_BM acceptor on the conformation of PTB7 donor in solution. The steady-state absorption spectra of varied PTB7 solution are recorded, and the results are shown in Figure 5. In PTB7 solutions (Figure 5a), the absorption spectra have some change with concentration. The amplitude ratios of 0′-0 and 1′-0 increase from 1.16:1 to 1.22:1, when the concentrations change from 7.8 × 10^−4^ mg mL^−1^ to 6.3 × 10^−2^ mg mL^−1^. It means the ordered aggregation degree of polymer is enhanced in solution. However, this ratio decreases to 0.95:1 at a concentration of 1 mg mL^−1^. It implies the disordered aggregation of donor polymer is dominant configuration in the high concentration solution. In Figure 5b, the absorption spectra of same PTB7 concentration values (7.8 × 10^−4^, 6.3 × 10^−2^, and 1 mg mL^−1^) in CB and CB:DIO = 97%:3% solvent were compared, respectively. The result shows that the absorption amplitude of PTB7 in CB:DIO is weaker than that in CB (A_PTB7, CB:DIO_ < A_PTB7, CB_), including high concentration and dilute concentration conditions. According to Beer-Lambert absorption law, A = *ε·b·c*, which A, ε, *b* and *c* represent absorbance, molar absorption coefficient, optical path and concentration of PTB7, respectively. In this work, a quartz cell with an optical path length of 1 mm was used. Therefore, a relationship can be determined, that is, *ε*_PTB7, CB:DIO_ < *ε*_PTB7, CB_. Here, we consider that *ε*_PTB7, CB:DIO_ is less than *ε*_PTB7, CB_ originating from DIO additive refinement the aggregation scale of PTB7 in solution [25]. Meanwhile, the amplitude ratios of 0′-0 and 1′-0 at same PTB7 donor concentration in CB and CB:DIO solvent are equivalent (Figure S2a). This indicates the DIO additive has little effect on the conformation of donor polymer in solution-phase. In Figure 5c, PTB7 in solution at various concentrations of 7.8 × 10^−4^, 6.3 × 10^−2^, and 1 mg mL^−1^ mixed with PC_71_BM acceptor also weaken the absorption of PTB7 donor, suggesting that PC_71_BM can reduce the aggregation scale of PTB7 in solution. Furthermore, the amplitude ratios of 0′-0 and 1′-0 in pure PTB7 solution and PTB7:PC_71_BM solution are unequal, implying that the PC_71_BM acceptor also plays an important role on the conformation of PTB7 donor (Figure S2b). Therefore, the morphology of active layers (Figure 4) can be regulated by the concentration of PTB7 donor, DIO additive and PC_71_BM acceptor.

### 3.5. Charge Generation and Lifetime in PTB7-Based Active Layers

**Figure 6:**
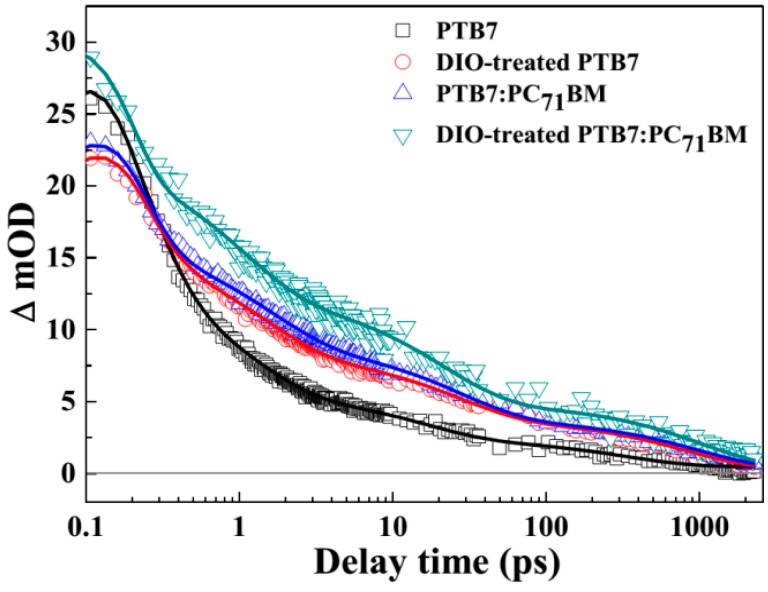
Kinetic curves of PTB7-based photoactive layers at a probe wavelength of 862 nm. All data were fitted by multi-exponential functions. All active layers were excited at 690 nm and the excitation photon fluence was 3.7 × 10^13^ photons·cm^−2^·pulse^−1^ at room temperature.

**Figure 7:**
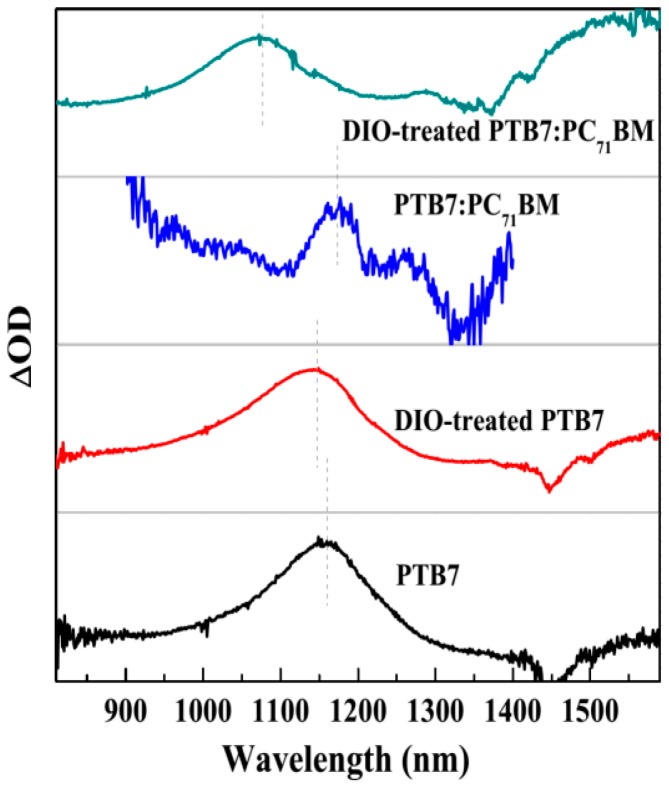
SEC spectra of PTB7-based active layers. All the differential spectra were taken as the difference between the spectra recorded with and without the applied potential. The spectral range of PTB7^•+^ was only exhibited in 810–1600 nm owing to experimental conditions.

To analyze charge generation and its lifetime characteristic in varied PTB7-based active layers, time-resolved spectroscopic measurements were carried out, and the TA spectra and kinetics data are shown in Figure S3, Figure 6 and Table 2, respectively. Our NIR spectral window is narrower than literature reported before [18], but the main conversion information of transient species can still be analyzed in our probe wavelength region (800–950 nm). At the same time, the absorption spectral characteristic of charged species (positive polaron) of PTB7 donor was obtained by the SEC measurement (cf. Figure 7). Hence, we probed the kinetic variation tendency of active layers at 862 nm. As seen from Figure 6 and Table 2, a multi-exponential function, y=y0+a1e−xτ1+a2e−xτ2+a3e−xτ3+a4e−xτ4, was used to fit the kinetic curves of excited PTB7-based photoactive layers. As for all active layers, four transient behaviors can be evidently identified. According to literature [26], the lifetimes of *τ*_1_, *τ*_2_, and *τ*_3_, are mainly related to exciton annihilation, exciton decays and charge transfer state decays, whereas the relative long-lived species of *τ*_4_ is attributed to positive polaron. Herein, we mainly focus on the population of charge generation and its lifetime in active layers. Obviously, the population (1.9 mOD) of positive polaron and lifetime (~332.9 ps) is lowest and shortest in PTB7 active layer. However, the population and lifetime of polaron are both increased while the PTB7 active layer treated with DIO or PC_71_BM-doped. This indicates more ordered PTB7 structure (Figure 4c,d) or D-A phase separation structure is beneficial to charge generation and charge transport. Moreover, as for DIO-treated BHJ PTB7:PC_71_BM active layer, compared with PTB7:PC_71_BM active layers, the population (4.4 mOD) and lifetime (> 1 ns) of polaron are improved, implying that appropriate D-A phase structure via DIO treatment has an important impact on the generation and lifetime of photogenerated charge.

### 3.6. Charge Transport Characteristic in PTB7-Based Active Layers

**Figure 8:**
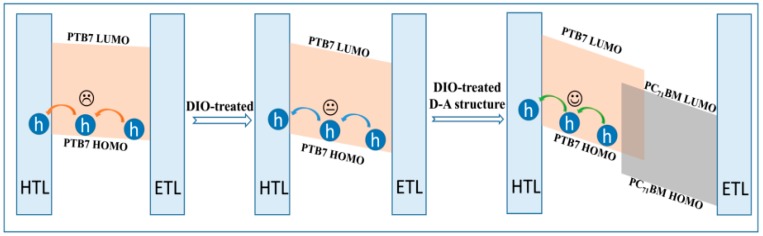
Schematic diagram of hole transport in PTB7, DIO-treated PTB7 and DIO-treated PTB7:PC_71_BM active layers controlled by energy band bending. Hole transport layer and electron transport layer are abbreviated as HTL and ETL, respectively.

The charged species hole of PTB7 donor in active layer plays a critical role in device performance. However, the morphology of active layer affects the hole generation and transport obviously [21]. Therefore, to obtain the characteristic spectra of hole in PTB7-based films, SEC measurements were carried out. The related results are shown in Figure 7. The steady-state absorption spectra of PTB7 is changed to that of PTB7^•+^ when it loses an electron under the condition of electric field. The main absorption bands are consisted of 800–1400 nm and beyond 1400 nm, which are similar with the absorption characteristic of P3HT^•+^ [27]. Here, we also identify the former as P2 transition absorption band, whereas the latter is referred as P1 absorption band. The P1 and P2 represent HOMO-1 → HOMO transition and HOMO → LUMO transition, respectively [28]. As for PTB7^•+^ in neat active layer, the maximum absorption peak of P2 is located at 1160 nm. However, the P2 peak has a slight blue-shift to 1145 nm after the PTB7 active layer treated with DIO additive. This indicates the energy gap of LUMO and HOMO becomes broaden, i.e., the DIO treatment of active layer results in downwards shifted HOMO levels and energy band bending [29], making the hole transport more efficiently (see Figure 8). Actually, the microcosmic ordered degree of PTB7 donor in the photoactive layer is greatly improved (seen Figure 3 and Figure 4) after the DIO additive treatment. Therefore, it is facilitative for charge transport in the donor phase. In BHJ PTB7:PC_71_BM active layer without DIO treatment, the P2 main peak has a slight red-shift to 1170 nm, presenting an upwards shifted HOMO levels. Hence, the transport property of hole in this bulk active layer is weaker than that in neat PTB7 film. Obviously, this is mainly due to the weakening degree of ordered aggregation of PTB7 phase, resulting from PC_71_BM phase [30]. Moreover, a shoulder peak presents at 1270 nm. It may be related to localized charge in BHJ PTB7:PC_71_BM active layer [31]. Interestingly, the P2 main peak (1070 nm) of BHJ PTB7:PC_71_BM active layer treated with DIO additive has a significant blue-shift effect, which has different values of 75 nm (76 meV) and 98 nm (97 meV) compared with DIO-treated PTB7 and PTB7:PC_71_BM active layers, respectively. This means that a large downwards shifted HOMO levels make the charge transport more efficient in DIO regulated D-A phase structure (Figure 8).

To further quantitively study the effect of DIO treatment and D-A structure on the charge transport properties of PTB7-based PSCs. The charge carrier mobility was estimated by using space charge limited currents model (SCLC), and the fabrication of devices and measurement conditions followed with literature [32]. The J-V characteristics of hole-only and electron-only devices are exhibited in Figure S4, and the values charge mobility are summarized in Table 3. PTB7 as a classical hole transport material shows the hole mobility and electron mobility are 2.60 × 10^−4^ cm^2^·V^−1^·s^−1^ and 6.96 × 10^−7^ cm^2^·V^−1^·s^−1^, respectively. Apparently, the hole mobility is three orders of magnitude higher than electron mobility in the PTB7 active layer. After DIO treatment, both the hole mobility and electron mobility are increased to 3.06 × 10^−4^ cm^2^·V^−1^·s^−1^ and 1.13 × 10^−6^ cm^2^·V^−1^·s^−1^. This is due to the improvement of the content of ordered aggregation in DIO-treated PTB7 active layer. In comparison, the electron mobility increased to 5.48 × 10^−5^ cm^2^·V^−1^·s^−1^, but hole mobility decreased to 5.60 × 10^−5^ cm^2^·V^−1^·s^−1^, in the case of PTB7 blend with PC_71_BM without DIO treatment. The reason is that the adding of high electron mobility material of PC_71_BM enhances the electron transfer and transport. Simultaneously, the morphology of PTB7 film is disturbed by PC_71_BM acceptor, resulting in lower hole mobility compared with PTB7 only active layer, which is in agreement with the spectral information (Figure 7). It also is found that balance of electron-hole mobility in the PTB7:PC_71_BM, and this should be helpful for the improving of J_sc_ and FF of PSCs accordingly. However, this balance is at a low charge mobility condition (10^−5^ level), hence, the device performance has not been optimized. In addition, compared to PTB7:PC_71_BM active layer, the hole mobility (5.60 × 10^−5^ cm^2^·V^−1^·s^−1^) and electron mobility (8.92 × 10^−4^ cm^2^·V^−1^·s^−1^) are enhanced again in the DIO-treated PTB7:PC_71_BM. The optimized charge mobility is due to the formation of efficient transport channels for hole and electon in donor phase and acceptor phase via DIO treatment. Therefore, the J_sc_, FF and PCE of PSCs have been improved by DIO treatment BHJ active layer.

## 4. Conclusions

In summary, the relevance among the solution state, solid state, photophysical property, and performance of PTB7-based photovoltaic devices with a variety of spectral and morphological characterization methods was studied. The reason of BHJ PTB7:PC_71_BM device with a PCE of 7.4% originates from the regulation of polymer morphology and D-A interface by using DIO additive. DIO refines the aggregation size of PTB7 donor in solution, and the optimized size can be remained into solid active layer. Moreover, PC_71_BM acceptor has an important effect on aggregation state including the aggregation scale and aggregation size of PTB7 donor in solution and solid states. DIO treatment and PC_71_BM phase lead to varied photophysical behaviors in PTB7 donor phase. The doped PC_71_BM phase contributes to exciton dissociation and electron transport. The population and lifetime of charged species can be improved in the DIO-treated active layers. The synergistic effect of DIO treatment and PC_71_BM phase can promote the band bending of PTB7 and increase the mobility of hole and electron. It is also found that the balance of electron-hole mobility only at high mobility condition is beneficial for the performance of PSCs. Therefore, our research results afford a deep understanding of relationship between D-A phase separation structure and device performance, and also provide a reference for the optimization of PSCs.

## Figures and Tables

**Table 1:** Photovoltaic parameters of PTB7-based polymer solar cells.

Active Layers	J_sc_ (mA/cm^2^)	V_oc_ (V)	FF (%)	PCE (%)	PCE-Max ^a^ (%)
PTB7	0.53 (±0.04)	0.64 (±0.02)	36.3 (±0.7)	0.13 (±0.01)	0.14
DIO-treated PTB7	0.73 (±0.10)	0.60 (±0.02)	32.4 (±0.6)	0.17 (±0.02)	0.19
PTB7:PC_71_BM	13.44 (±0.18)	0.70 (±0.01)	48.5 (±0.5)	4.52 (±0.22)	4.74
DIO-treated PTB7:PC_71_BM	15.45 (±0.16)	0.71 (±0.02)	64.7 (±0.3)	7.30 (±0.11)	7.41

^a^ The PCE-max are the highest values in 24 devices of each active layer.

**Table 2:** Decay time constants (*τ*, ps) and amplitude values (a, mOD) derived from multi-exponential curve fitting of the kinetics for PTB7-based photoactive layers.

Active Layers	Fitting Parameters and Apparent Lifetimes							
	*a*_1_	*τ*_1_	*a*_2_	*τ*_2_	*a*_3_	*τ*_3_	*a*_4_	*τ*_4_
PTB7	28.5	0.17 ± 0.04	8.1	1.7 ± 0.4	3.2	15.7 ± 1.5	1.9	332.9 ± 133.8
DIO-treated PTB7	23.6	0.12 ± 0.03	7.7	1.4 ± 0.2	4.5	25.8 ± 5.5	3.4	815.9 ± 141.2
PTB7:PC_71_BM	31.4	0.10 ± 0.03	7.4	1.5 ± 0.2	5.2	27.2 ± 4.9	3.6	995.7 ± 158.2
DIO-treated PTB7:PC_71_BM	18.3	0.24 ± 0.01	10.1	1.0 ± 0.2	7.5	20.0 ± 4.3	4.4	1054.2 ± 235.1

**Table 3:** The averaged mobility parameter values (*μ*) of charge in each PTB7-based polymer solar cells base on 24 devices.

Charge Carrier	PTB7	DIO-Treated PTB7	PTB7:PC_71_BM	DIO-Treated PTB7:PC_71_BM
Hole (cm^2^·V^−1^·s^−1^)	2.60 (±0.1) × 10^−4^	3.06 (±0.1) × 10^−4^	5.25 (±0.15) × 10^−5^	5.60 (±0.15) × 10^−5^
Electron (cm^2^·V^−1^·s^−1^)	6.96 (±0.1) × 10^−7^	1.13 (±0.1) ×10^−6^	5.48 (±0.1) × 10^−5^	8.92 (±0.1) × 10^−4^

## Supplementary Materials

The following are available online at http://www.mdpi.com/2073-4360/10/3/332/s1.
