# Supplementary material for: Appropriate Donor-Acceptor Phase Separation Structure for the Enhancement of Charge Generation and Transport in Polymer Solar Cells
Source: Polymers (Basel). 2018 Mar 18;10(3):332. doi: 10.3390/polym10030332 (PMC6414981; doi:10.3390/polym10030332)
Supplement: Supplementary file 1 [file polymers-10-00332-s001.pdf]

Supplementary Information

Appropriate Donor-Acceptor Phase Separation Structure for the Enhancement of Charge Generation and Transport in PTB7-based Polymer Solar Cells

Dayong Zhang[1,2], Rong Hu[2,*], Jiang Cheng[2], Yuqiang Chang[3], Mingming Huo[4], Junsheng Yu[1], Lu Li[2, *], and Jian-Ping Zhang[3]

[1]*State Key Laboratory of Electronic Thin Films and Integrated Devices, School of Optoelectronic Science and Engineering, University of Electronic Science and Technology of China (UESTC), Chengdu 610054, P. R. China*

[2]*Research Institute for New Materials Technology, Chongqing University of arts and sciences, Chongqing 402160, P. R. China*

[3]*Department of Chemistry, Renmin University of China, Beijing, 100872, P. R. China*

[4]*Qingdao Research Center for Advanced Photonic Technologies, Laser Research Institute, Shandong Academy of Sciences, Qingdao 266100, P. R. China*

S1. Steady-state absorption spectra of $PC_{71}BM$ solutions.

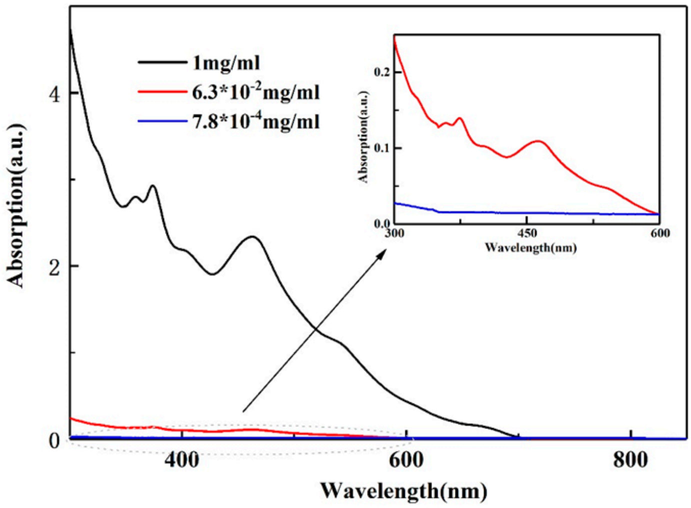

Figure S1. Steady-state absorption spectra of PC71BM solution at different concentrations.

S2. Effects of DIO and $PC_{71}BM$ on aggregation state of PTB7 in solution

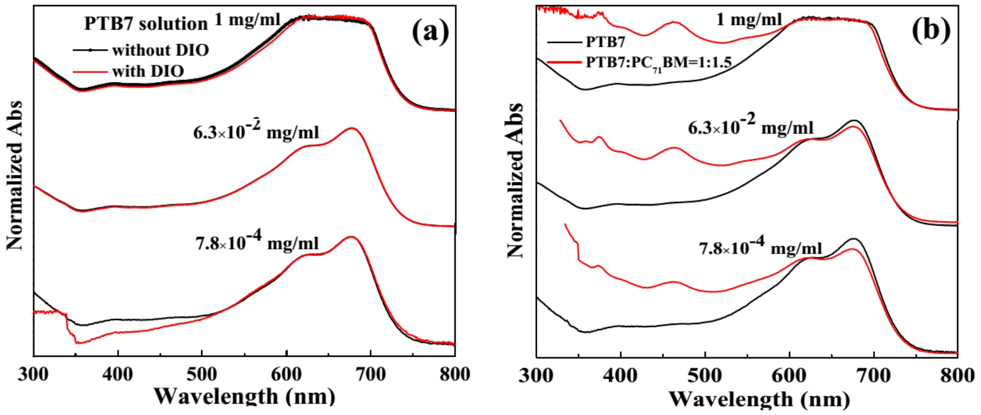

Figure S2. Steady-state absorption spectra of PTB7 solution and mixed PTB7:$PC_{71}BM$ solution at varied concentration conditions. (a) represents PTB7 dissolution in CB solvent or CB:DIO=97%:3% solvents. (b) represents PTB7 or PTB7:PC71BM=1:1.5 dissolution in CB solvent. All spectra were normalized at 623 nm.

S3. Transient absorption spectra of PTB7-based active layers

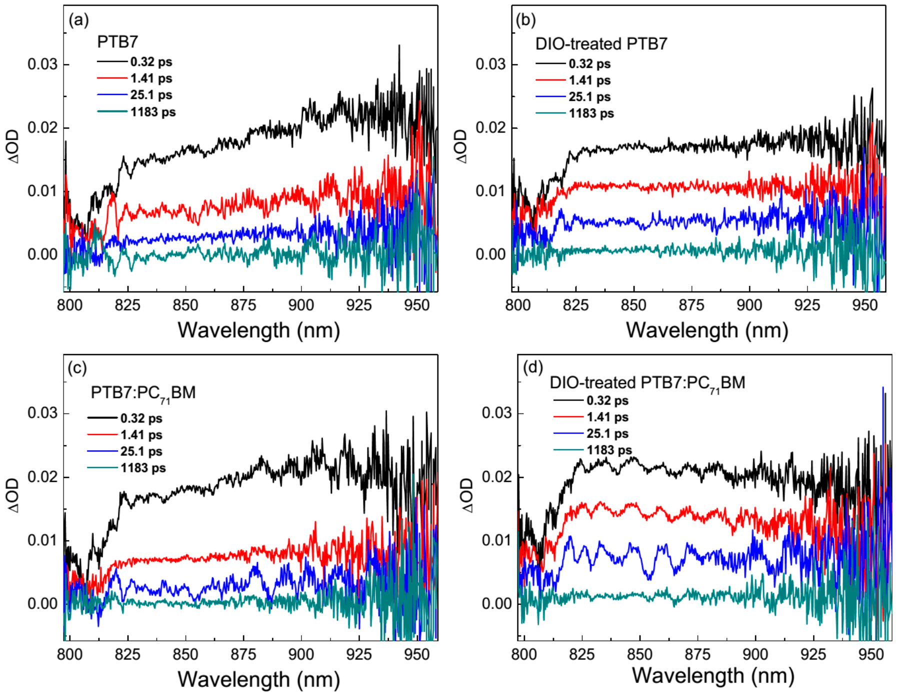

Figure S3. Transient absorption spectra of PTB7 (a), DIO-treated PTB7 (b), PTB7:$PC_{71}BM$ (c), and DIO-treated PTB7:$PC_{71}BM$ (d). All active layers were excited at 690 nm and the excitation photon fluence was 3.7×10^{13} photons·cm^{-2}·$pulse^{-1}$ at room temperature.

S4. Estimation of hole and electron mobility in PTB7-based active layers by SCLC model.

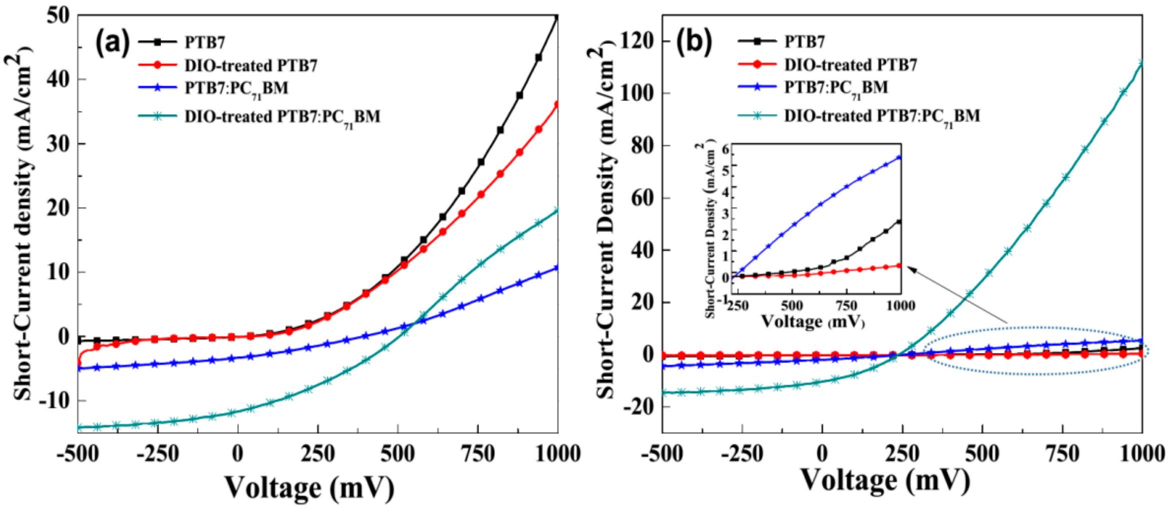

Figure S4. (a) J-V characteristics of hole-only devices based on varied active layers. (b) J-V characteristics of electron-only devices based on varied active layers. The inset: Enlarged J-V characteristics from 250 to 1000 nm. The hole mobility and electron mobility in the hole-only and the electron-only devices can be calculated by Mott-Gurney law as equation [S1]: $J=9/8\varepsilon\varepsilon_0V^2/d^3$ where ε is the relative permittivity of polymer assumed to be 3, and ε0 is the vacuum dielectric constant of 8.85×10^{-12} F/m. V is the voltage, and d is the thickness of active layer.

REFERENCES

S1. Wang, H.; Huang, J.; Xing, S.; Yu, J. Improved mobility and lifetime of carrier for highly efficient ternary polymer solar cells based on tips-pentacene in PTB7: $PC_{71}BM$. *Organic Electronics* **2016**, *28*, 11-19.

Corresponding Authors:

E-mail: hurong_82@cqwu.edu.cn (R. Hu), lli@cqwu.edu.cn (L. Li).
